# The role and impact of research positions within health care settings in allied health: a systematic review

**DOI:** 10.1186/s12913-016-1606-0

**Published:** 2016-08-05

**Authors:** Rachel Wenke, Sharon Mickan

**Affiliations:** 1 Allied Health, Ground Floor D block, Gold Coast University Hospital, 1 Hospital Boulevard, Southport, QLD Australia 4215; 2 School of Allied Health Sciences, Griffith University, Gold Coast, Australia; 3 Allied Health, Gold Coast Hospital and Health Service, Southport, QLD Australia

## Abstract

**Background:**

Embedding dedicated research positions within healthcare settings is a potential strategy to build allied health research capacity, with different health care organisations investing in such positions. The aim of this review was to gather evidence regarding the nature of the role of the research position in allied health professional (AHP) healthcare settings and the impact that these positions have on building research capacity.

**Methods:**

A systematic review was undertaken searching eight databases (Medline CINAHL, Cochrane, OTSeeker, Speechbite, PEDro, Web of Science, and Proquest) using English language restrictions. Both authors independently screened abstracts, reviewed full-text articles, extracted data and performed quality assessments using the Mixed Methods Appraisal Tool. Studies were included that reported the evaluation and/or components of the role of a dedicated research position with AHPs in any healthcare setting. A thematic analysis approach was used to synthesise findings.

**Results:**

A total of 360 abstracts were initially screened, with 58 full text articles being reviewed. Eight unique studies were included in the thematic analysis clarifying either the nature of role of the research position (*n* = 7) or impact of the position (*n* = 4). Studies included mixed methods (*n* = 3), descriptive case study (*n* = 4), and observational (*n* = 1) designs. The majority of studies reported the research positions to provide academic support to individual clinicians and their teams, while developing their own research projects. Other studies reported support for research capacity building at a service and organisational level. Positive changes from these research positions was reported via increased individual research skills and participation and research outputs, improvements in research culture, attitudes and team and organisational level skills.

**Conclusion:**

Emerging evidence suggests that research positions embedded within healthcare settings can influence individual and team based research skills and research participation of AHPs. Future research is needed to further investigate the sustainability of changes arisen from research positions and what mechanisms of the positions have the greatest impact. Healthcare managers should consider how to support potential components of the research position roles identified in the literature, as well consider evaluating their impact on research capacity, cultural and attitudinal changes of AHP staff in addition to traditional research metrics.

**Electronic supplementary material:**

The online version of this article (doi:10.1186/s12913-016-1606-0) contains supplementary material, which is available to authorized users.

## Background

Health professionals working within a healthcare organisation are in an excellent context for carrying out research due to their close access to patients and opportunity for clinically driven research questions [1]. Benefits associated with research engagement within healthcare organisations are also extensive and may include reduced staff turnover, increased productivity and efficiency and lower patient mortality [2–4]. Despite these benefits, many clinicians working within healthcare organisations lack the skills, confidence and opportunity to undertake research. These challenges have been reported to be particularly prevalent within allied health [1, 5–7], a workforce comprised of a number of diverse professions who work closely with medical and nursing staff. Allied health professions comprise the third largest workforce within health care and include physiotherapy, occupational therapy, social work, speech pathology, psychology, dietetics, podiatry, and radiography, among others. Healthcare organisations have placed increased priority on helping promote allied health professionals to undertake research through a variety of initiatives aiming to build research capacity [8–10]. Embedding dedicated research positions within healthcare settings is one such strategy [10–12]. Indeed, the past decade has seen different Australian and United Kingdom health care organisations investing in such positions in order to promote research capacity of health professionals [11, 13].

Various terminologies have been used to describe dedicated research positions employed within healthcare settings. Such terms include “research facilitator” [14, 15] “research fellow” [16, 17] “research lead” [18] or “clinical academics” [13]. Such positions may be funded solely by a healthcare organisation or jointly funded in partnership with a university institution [11, 13, 19]. Unlike research academics, these dedicated research positions are embedded within a healthcare setting as opposed to exclusively working within a university institution.

A number of observational studies using survey and interview, as well as editorial pieces have identified that embedding these research positions within healthcare settings can be an enabler to allied health research and should be advocated for by health organisations [10, 17–19]. For example, Brauer et al., [19] described the investment of co funded research positions between healthcare organisations and university institutions as a potential means for “bridging the gap between clinicians and research expertise”. Interviews of senior allied health managers within an Australian health care setting also described that dedicated research positions, as well as other infrastructure contributed to a positive impact on allied health research and were important contributors to motivating staff and providing opportunities for career pathways in research as well as attracting experienced researchers to drive research [10]. A survey of Australian physiotherapists with PhDs also described potential benefits of dedicated research positions within clinical healthcare settings. Respondents indicated that additional joint academic-clinical research appointments would enhance physiotherapy research careers by facilitating collaborations and clinically-relevant projects, as well as fostering excellence and improving job security [20].

Despite the potential benefits, only a limited number of allied health teams have access to a dedicated research position to provide support. Currently, the majority of data pertaining to the prevalence of such positions comes from Australia. A recent survey of 520 allied health professionals across all Victorian hospitals revealed that approximately one third of respondents had access to a co-located research position within their workplace [18]. A survey of physiotherapy departments across Australia reported that over a quarter had a dedicated research position [21], with the majority being within metropolitan hospitals. The need for research fellow positions within rural settings to promote research capacity building of allied health was additionally highlighted in a recent Australian study [17]. In order to justify ongoing and additional investment in dedicated allied health research positions, there needs to be greater understanding of the nature of the role and the impact of these research positions in building allied health research capacity and engagement. To address this gap, we aimed to answer the following questions; (1) What is the role of the dedicated allied health research positions in healthcare settings? and (2) What is the impact of allied health research positions in building research capacity in healthcare organisations?

## Methods

We conducted a systematic review and thematic analysis to synthesise and appraise current evidence relating to dedicated research positions within allied health healthcare settings.

### Search methods

In collaboration with the two authors, a research librarian developed a search strategy in eight electronic databases (Medline (Ovid), CINAHL (Ebsco), Cochrane Central Register of Controlled Trials (CENTRAL) latest issue, Web of Science (Thomson Reuters), and Proquest (Dissertations and theses global), and OTSeeker, Speechbite, PEDro using English only restrictions. Database searches were run from inception to August 31 2015, with the exception of the latter three databases which were run from inception to September 2015. Reference lists of included articles were additionally reviewed. Terms and synonyms related to capacity building, research personnel and allied health were used. An example of the search strategy used in Medline is found in Additional file 1 and was used to search the other databases.

### Study inclusion and exclusion criteria

We used two levels of inclusion criteria dependant on the research question being answered. To understand the impact of allied health research positions, we included experimental or quasi-experimental studies (including non-randomised, group and single subject design) which reported the evaluation of a dedicated research position which provided research support to allied health professionals in any healthcare setting. For the purposes of this review, allied health professionals included the following professions: physiotherapy, occupational therapy, social work, speech pathology, psychology, dietetics, podiatry, and radiography and pharmacy. To clarify the nature of the role of the dedicated research positions in allied health healthcare settings, we also included descriptive studies, which reported the application of a dedicated research position supporting allied health research professionals. We excluded studies where research positions were based exclusively within an academic setting, where they did not include allied health professionals, or were unavailable in English.

### Study selection

**Fig. 1:**
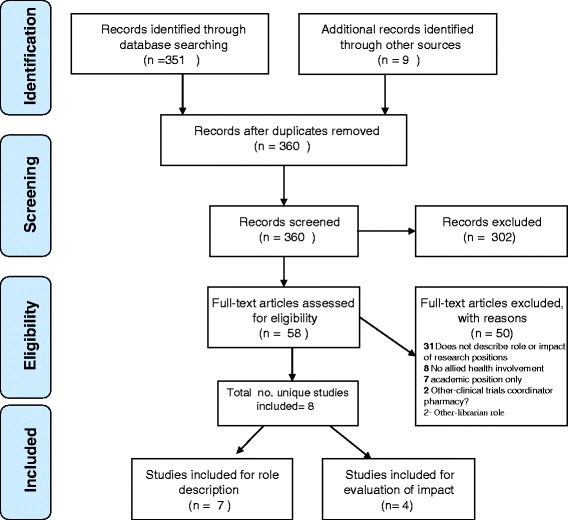
Flow diagram of process to identify eligible articles

Both authors independently screened all titles and abstracts from database searches and grey literature sources, and discrepancies were resolved with a consensus meeting. Full-text articles were also obtained if eligibility could not be determined from the title or abstract. Both authors independently assessed study eligibility from the full text and disagreements were resolved by discussion and consensus agreement. Where clarification was required, one of the authors (RW) contacted the study authors to request the relevant information. Reasons for exclusion of studies were documented as shown in Fig. 1.

### Quality assessment and data extraction

The assessment of risk and bias of the included empirical studies was evaluated by both authors using the Mixed Methods Appraisal Tool or MMAT [22]. This tool was designed to appraise studies with diverse designs including qualitative, quantitative and mixed methods research [23]. Specifically, studies were scored on the criteria summarised in the footnote of Table 2 depending on study design.

A data extraction form was developed by the review authors to include information pertaining to study design and location, participants, (i.e., health profession, number) description of research position role (i.e., who provided support to, funding of role, duration of role, setting), and specific tasks/activities of role. For studies that evaluated the impact of the research positions, data was extracted to describe the nature of the research position (i.e., full time/part time, level of experience, profession), outcome measures and time points, results for each quantitative outcome and time of assessment specified, and qualitative results. Disagreements were resolved by discussion and consensus with the two authors.

### Data analysis and synthesis

Following data extraction, both authors conducted a thematic analysis to identify key components of the role of the dedicated research position. This involved one of the authors (RW) coding the data which referred to the nature of the roles or activities that the research position undertook into descriptive themes. A second author (SM) then checked the data for reliability of category formation. A separate thematic analyses was undertaken to synthesise the evidence describing the impact or outcomes of the research positions using the same process.

## Results

### Study characteristics

A total of 8 articles were included in the review. These included mixed methods (*n* = 3), descriptive case study (*n* = 4), and observational (i.e., cross sectional survey) (*n* = 1) designs. Seven of these studies met the inclusion criteria (see Fig. 1) for answering the question relating to the role of the research positions. Four studies met the inclusion criteria for answering the question relating to impact of the research positions. Study characteristics including participant description and nature of research position role in the study are shown in Table 1. Staff in these research positions predominately supported allied health professionals, with some additionally supporting medical and/or nursing staff. Type and number of participants in each study varied, however participants were predominately allied health professionals with the exception of two studies which also included medical [24] and nursing professionals [14]. Study origin included Australia (*n* = 3), United Kingdom (*n* = 3), New Zealand (*n* = 1) and USA (*n* = 1).

**Table 1:** Study characteristics

Study	Study design	Described role or impact?	Participants description	Geographical location	Participant no.	Nature of research position role in study				
						Location/Setting of role	Profession of role	Provided support to	Funding of role	Duration of role
Davila et al, 2006 [25]	Descriptive case study	Role	Psychologist	Boston USA	1 research facilitator position	Academically affiliated medical centre	Psychologist	psychology, psychiatry, pulmonology, primary care	Grant funded	6 years plus
Hulcombe et al.,2014 [11]	Descriptive case study	Role	Early career researchers to professors across AHPs	Queensland, Australia	21 positions across 8 health services.	Queensland government health services	Variable AHPs	AHPs	3 of 21 positions funded by government, others co-funded with universities	unclear
Janssen et al., 2013 [15]	Mixed methods	Role & impact	Physios	New Zealand	22 physios, 3 managers, 1 research facilitator	Rehabilitation hospital	Physio	Physios and their managers	Burwood academy of independent living and University of Otago	1 year full time
Perry et al., 2008 [14]	Mixed methods	Role & impact	Nurses, midwives, AHPs and managers	London, UK	98 completed questionnaires, 19 senior managers interviewed	East London NHS Trust	Nurse	Nurses, AHPs	Local funding	5 years, full time
Ried et al., 2007 [9, 16]	Mixed methods	Role & Impact	AHPs	Adelaide, South Australia	3 research fellows	Primary health care	unclear	not clear - fellowship for personal research	Government funding	0.2-0.5 FTE for 1 year
Reid et al., 2011 [24]	Descriptive case study	Role	radiographers, undergraduates, registrars, radiologists	Norfolk, UK	unclear	University teaching hospital	Radiographer	radiology department	Government funding	5 years
Whitworth et al., 2012 [26]	Descriptive case study	Role	SLPs	North East England	unclear	Primary care NHS North of Tyne area	unclear	SLPs	Partnership between university and NHS	1 year (initially)
Williams et al., 2015 [18]	Observational (cross sectional survey)	Impact	AHPs	Victoria, Australia	520 completed surveys	Victorian health care	20 different AHP	AHPs	unclear	unclear

*Physio* physiotherapist, *SLP* speech-language pathologist, *FTE* Full time equivalent, *AHP* allied health professional, *NHS* National Health Service

Research positions described in the included studies were embedded in a variety of health settings including hospitals and a medical centre. Most research positions were filled by allied health staff and one study included a nurse [14]. Where specified, funding of positions was generally either from solely the government health care provider (*n* = 3), or a partnership between the government and a university (*n* = 3). The duration of the research position roles varied from 1 to 6 years, and ranged from part time to full time.

### Risk of bias

Quality assessment was undertaken using the Mixed Methods Appraisal Tool on all empirical studies. The studies which were descriptive in nature and did not include any formal evaluation were unable to meet the preliminary criteria for the Mixed Methods Appraisal Tool (i.e., did not have a clear qualitative and/or quantitate research questions) and therefore could not be appraised. Results are found in Table 2, which describes largely positive scores for qualitative, quantitative and mixed methods measures. While some studies were not clear about the quality of aspects of qualitative or qualitative analysis, most were consistent in their integration of data sets for the mixed methods interpretations.

**Table 2:** Outcome measures for evaluation studies and quality assessment

	Outcome measure		Mixed methods appraisal tool score			
	Quantitative (and time point)	Qualitative	Qualitative	Quantitative	Mixed methods	TOTAL score (out of maximum)
Participant interviews						
					5.1 – Y	8/11
Janssen et al., 2013 [15]	Edmonton Research Orientation Survey (EROS)		1.1-Y	3.1-Y	5.2- Y	
	(pre, post,12 months follow up)		1.2- Y	3.2-Y	5.3- Y	
	VAS-Confidence & motivation towards research		1.3-Y	3.3- N		
			1.4-Unclear	3.4- Unclear		
Perry et al., 2008 [14]	Service user survey (post)	Senior manager interviews			5.1 –Y	7/11
	Audit of requests for support		1.1-Y	4.1- Unclear	5.2- Y	
			1.2- Y	4.2-Unclear	5.3- Y	
			1.3-Y	4.3- Y		
			1.4-Unclear	4.4- N		
Ried et al., 2007 [9, 16]	Research Spider (pre-post)	Participant interviews	1.1-Y	4.1- Y	5.1 – Y	8/11
			1.2- Unclear	4.2 –Y	5.2- Y	
	publications, personal higher degree enrolment		1.3-Unclear	4.3-Y	5.3- Unclear	
			1.4- Y	4.4-Y		
Williams et al., 2015 [18]	Research Capacity and Cultlure questionnaire	n/a		4.1-Y		3/4
	Self-report of research activity undertaken			4.2- Y		
				4.3- Y		
				4.4-Unclear		

N.B. Qualitative 1.1. Are the sources of qualitative data relevant to address the research question?1.2. Is the process for analyzing qualitative data relevant to address the research question? 1.3. Is appropriate consideration given to how findings relate to the context1.4. Is appropriate consideration given to how findings relate to researchers’ influence, ? 3. Quantitative nonrandomized 3.1. Are participants recruited in a way that minimizes selection bias? 3.2. Are measurements appropriate (clear origin, or validity known, or standard instrument; and absence of contamination between groups when appropriate) regarding the exposure/intervention and outcomes? 3.3. In the groups being compared are the participants comparable, or do researchers take into account the difference between these groups? 3.4. Are there complete outcome data (80 % or above), Quantitative descriptive 4.1. Is the sampling strategy relevant to address the quantitative research question? 4.2. Is the sample representative of the population understudy? 4.3. Are measurements appropriate (clear origin, or validity known, or standard instrument)?4.4. Is there an acceptable response rate (60 % or above)? 5. Mixed methods 5.1. Is the mixed methods research design relevant to address the research questions? 5.2. Is the integration of qualitative and quantitative data (or results*) relevant to address the research question (objective)? 5.3. Is appropriate consideration given to the limitations associated with this integration, e.g., the divergence of qualitative and quantitative

### Components of research position role

The role of allied health research positions was summarised across three main themes: (1) provision of academic support to individual and/or teams, (2) development of own research and (3) service level/organisational support (see Table 3). The majority of studies described the role of the research position as providing academic support to individuals and/or teams at multiple stages throughout the research cycle [11, 14, 15, 24–26]. Specific mention was made of supporting individuals to get started in research, obtain funding, coordinate and disseminate projects. Other tasks included helping to establish collaborations, providing education and training, and facilitating research utilisation.

**Table 3:** Summary of components of research position role

Components of Research Role	Davila et al., 2006 [25]	Hulcombe et al., 2014 [11]	Janssen et al., 2013 [15]	Perry et al., 2008 [14]	Ried et al., 2007 [9, 16]	Reid et al., 2011 [24]	Whitworth et al., 2011 [26]
Provide academic support to individuals and/or teams							
Getting started in research				x			
Obtain funding for research				x			
Disseminating research (writing for publication)				x			
Ongoing support of projects (e.g., mentoring/ encouragement)			x	x		x	
Assist groups in steps of research (e.g., ethics, conducting literature review, grant writing)			x			x	x
Help establish collaborations and networks			x	x			
Education and training	x	x	x	x		x	
Support research utilization			x	x			
Develop own research							
Undertake/develop specific research projects/streams	x	x			x	x	x
Supervise students (i.e., research higher degree) or staff	x	x					
Conference presentations		x					
Prepare publications		x			x		
Attract research grant funding		x				x	
Service level/organisational support							
Strategy development				x			
Establish service level and study agreements						x	
Establish database of research activities				x		x	
Establish research conferences				x			
Leadership and collaboration through networks and governance		x		x			
Develop research culture/ promote research activity				x		x	
Produce annual report						x	
Other non-research tasks							
Clinical work	x						
Managing events							x

The majority of studies described ways in which the individual who occupied the research position developed their own research projects (*n* = 5) [11, 16, 24–26]. Associated with this task, they also supervised students, disseminated research presentations and publications and attracted research funding. Service level and organisational support was identified as an important role of the research position in three of the included studies [11, 14, 24]. Examples of this aspect of the role included strategy development, establishing databases of and promoting research activity, providing leadership through networking and collaboration, and developing research culture.

### Impact of the research position

The impact of the research position on allied health research capacity can be broadly summarised across four themes (see Table 4). These include (1) increased individual research skills and participation, (2) increased research activity (3) improved research culture and attitudes and (4) increased team and organisational level skills,. Improvements have been reported in three studies for individual research skills and participation in research. Participants reported increased self- confidence in dissemination, funding and data collection [16] as well as increased individual involvement in these activities [14, 18]. These improvements can be ascribed to the individual in the research position Ried et al. [16] and to the allied health clinical staff they support.

**Table 4:** Impact of Research Positions

	Study			
Reported area of change	Janssen et al. 2013	Perry et al. 2008	Ried et al., 2007	Williams et al., 2015
Individual research skills or participation				
Writing/Dissemination	n/a	n/a	Greater self-reported competence in writing research protocol, publishing research, writing & presenting a research report	More involvement in writing publications, presentations and reports for participants who had access to RP
Funding	n/a	Increased no. staff applying for research funding	Greater self-reported competence in applying for research funding	Increased applications for research funding for participants who had access to RP
Data collection an analyses	n/a	n/a	Greater self-reported competence in Using qualitative and quantitative research methods. No change to interpreting data	Increased involvement in data collection for participants who had access to RP
Other individual skills	n/a	n/a	Greater confidence in critically reviewing literature, finding relevant literature , generating research ideas	
Research activity and output	n/a	Qualitative reports of increasing numbers of research related activities (increased numbers of staff undertaking research training, participating in research, applying research findings to practice)	Two RPs had prepared at least one manuscript for publication, one fellow applied for PhD scholarship	Increased research activity reported by organisations with RPs
Research culture & attitudes towards research	All four teams showed increased orientation towards research. Improved confidence in 3 out of 4 teams	Improved research culture (practical, informational, and inspirational support, more aware of uses of research, growing ground-swell of interest and enthusiasm about research) Potential service gains (development of patient care, best practice services)	n/a	n/a
Team and organisational level research skills	n/a	n/a	n/a	All items of Research Capacity and Culture tool for team and organisational level were higher in organisations with RP

*= *RP(s)* research position(s)

Three of the four included studies also reported increases in research activity as a result of the research positions [14, 16, 18]. This included an increased number of staff undertaking research related training and activities, as well as evidence of more traditional research outputs (i.e., manuscript preparation). Changes in research culture and attitudes towards research were also reported in two studies [14, 15]. Physiotherapists in New Zealand reported improved orientation towards research and confidence, which was measured by perceived value of research, involvement in research, being at the leading edge and using evidence based practice [15]. Clinical staff in England reported increased interest and enthusiasm for research, improvements in patient care and high levels of satisfaction with the swift and effective responses from the research position [14].

Williams et al., [18] reported the positive impact of a research position on team and organisational level research skills across all 18 and 19 items respectively of the research capacity and culture tool. Reported research skills using this questionnaire were significantly higher in organisations that had a research position. Examples of these items that were rated higher in organisations that included a research position included having adequate resources to support staff training, engaging with external partners (e.g., universities), supporting peer-reviewed publications, and promoting research activities which are relevant to practice.

## Discussion

There is developing consensus in the literature about the role and impact of allied health research positions, within health care organisations. Individuals in these roles are commonly reported to provide academic support to individual clinicians and their teams, while developing their own research projects. They have also offered support for research capacity building at a service and organisational level. Positive impact from these research positions has been reported via increased individual research skills, participation and research outputs. Improvements in research culture, attitudes and team and organisational level skills have also been documented.

Furthermore, study authors have emphasised additional factors that may positively support and build upon the reported impact of these research positions. There is recognition that managers can positively influence the organisational culture to actively support research engagement [14]. Research can be linked at a team and organisational level with education, professional development, service improvement and practice development activities [14]. Individually, research tasks and objectives can be embedded within job descriptions, performance development and appraisal programmes [15]. There can be motivational and attitudinal benefits from developing a critical mass of clinicians who are engaged with research [15]. Overall, it appears that specific strategies should be developed and tailored for individual and team profiles within organisations [18]. Further, when research posts are integrated between clinical and academic environments there can be a focus on translating new research evidence into clinical care improvements [11].

Findings of the review highlight the potential value of research positions being embedded within healthcare organisations. When considering the addition of a research position within a healthcare setting, stakeholders may want to consider that the role is supported to undertake a diverse range of activities within their organisation and at different levels. This may include finding the balance between undertaking their own research as well as supporting the development of research skills in and engagement of individuals and teams. Based on findings of the potential impacts of the research positions, performance evaluation of these positions should consider not only traditional academic research metrics (i.e., publications, grant funding) but also how the role has supported individual and teams in their research development and engagement, as well as potential changes in attitude and research culture.

A significant limitation of this systematic review is noted in the widespread use of self-reported surveys and participant interviews. While there has been rigour in the reporting and analysis of this data, there is a high likelihood of a positivity bias by participants who are engaged and enthusiastic about research. Future research should aim to understand and maximise how these research positions facilitate the positive impact on research capacity building. Evaluations should consider outcome measures that evaluate changes to individual research skills, research outputs, organisational/service outcomes and research culture and attitudes, as these areas were found to be potential areas of impact. It is recommended that future research also considers a mixed methods research design, incorporating collection of both quantitative and qualitative data to assist in the understanding of the depth of the impact and the active ingredients of the role. Future research should seek to understand how to best to support and enhance the impact of these positions through leadership and team based interventions.

## Conclusion

There is some early evidence to suggest that research positions embedded within healthcare settings can influence individual and team based changes within allied health. The literature provides some potential areas that stakeholders may wish to consider when implementing research positions within their healthcare organisation, and how to evaluate their impact. Future research should investigate the longevity and spread of changes arisen from research positions and more specifically what aspects or mechanisms of the positions within their context lead to the greatest influence on research capacity building of allied health individuals and teams.

## Abbreviations

AHP, allied health professional; MMAT, Mixed methods appraisal tool

## Additional file

Additional file 1: Search strategy (DOC 22 kb)
