# Supplementary material for: The role and impact of research positions within health care settings in allied health: a systematic review
Source: BMC Health Serv Res. 2016 Aug 5;16:355. doi: 10.1186/s12913-016-1606-0 (PMC4974741; doi:10.1186/s12913-016-1606-0)
Supplement: Additional file 1: — Search strategy (DOC 22 kb) [file 12913_2016_1606_MOESM1_ESM.doc]

**Additional file 1:**

Search strategy used in Medline:

An example search strategy used in Medline is found below:

1     Capacity Building/ (1048)

2     (building adj2 capacity).tw. (3070)

3     or/1-2 (3670)

4     exp Research/ (470070)

5     Research Personnel/ (12048)

6     (research adj3 (position* or team* or coordinat* or activit* or participat* or skill* or success* or capacit* or engag*)).tw. (25758)

7     or/4-6 (491452)

8     employment/ or employment, supported/ (38739)

9     Job Description/ (10613)

10     "Task Performance and Analysis"/ (25476)

11     Professional Role/ (9183)

12     Professional Competence/ (21132)

13     or/8-12 (102443)

14     exp Allied Health Personnel/ (43690)

15     exp Allied Health Occupations/ (45586)

16     allied health.tw. (4793)

17     or/14-16 (91501)

18     3 and 7 and 17 (42)

19     7 and 13 and 17 (494)
